# Impact of the COVID-19 Pandemic on Adult Mental Health

**DOI:** 10.12669/pjms.36.COVID19-S4.2756

**Published:** 2020-05

**Authors:** Imran Ijaz Haider, Farah Tiwana, Sania Mumtaz Tahir

**Affiliations:** 1 Prof Dr. Imran Ijaz Haider (FRCPsych, MRCPsych, DPM (UK), Professor of Psychiatry & Behavioural Sciences, Fatima Memorial Hospital, Shadman, Lahore, Pakistan; 2 Farah Tiwana MSc. (UK), Clinical Psychologist, Department of Psychiatry & Behavioural Sciences, Fatima Memorial Hospital, Shadman, Lahore, Pakistan; 3 Dr. Sania Mumtaz Tahir MBBS. Post Graduate Resident Department of Psychiatry & Behavioural Sciences, King Edward Medical University, Fatima Memorial Hospital, Shadman, Lahore, Pakistan

**Keywords:** COVID-19, Mental Health, Pandemic Impact

## Abstract

The outbreak of the Novel Coronavirus (COVID-19) in December 2019 has progressed to the status of a global pandemic, with countries across the seven continents adversely affected and the number of human cases exceeding two million. With no available vaccine, the treatment is primarily symptomatic for those affected and preventative for those at risk. Most countries have taken action to curtail the spread of COVID-19 through measures such as lockdowns, social distancing and voluntary self-isolation. Whilst necessary, such measures and the disease itself, may have an adverse impact on mental health. In view of research from previous pandemic crises, it is known that such situations are likely to increase stress levels and have negative psychiatric effects. The impact is likely to be felt by the general public, sufferers of COVID-19, their families and friends, persons with pre-existing mental health conditions and healthcare workers.

## Mental Health Impact of COVID-19

Coronaviruses are a group of viruses that may cause illness in humans and animals.^1^ Humans present with mild to severe respiratory diseases when affected by coronaviruses, the most recently discovered of which is COVID-19.^2^ The outbreak of COVID-19 human cases occurred in late December 2019 in the Wuhan province of China.^3^ Since then, the disease has spread globally achieving pandemic status, affecting countries in Europe, the U.S, the U.K, Asia, the Middle East and North Africa^4^, with 7025 confirmed cases in Pakistan as of 22/04/20.^5^ At present, there is no COVID-19 vaccine, but symptomatic treatment is possible. Numerous countries, including China, and various organisations, including the Food & Drug Authority (FDA) in the USA, are working on drug development. However, even if a drug is developed, there remain concerns about the level of production that will be required, and whether it will be accessible to the majority of the world’s population.^6^

The current global pandemic inevitably has consequences for mental health, as shown through previous health crises. For instance, the SARS (Severe Acute Respiratory Syndrome) outbreak in Hong Kong in 2003 has been described as a “mental health catastrophe”^7^, with long-term psychiatric morbidities such as PTSD (Post-Traumatic Stress Disorder) and Depression^7^. The development of psychiatric morbidity was mediated by factors such as social support and disease-related worry.^8^

### Impact on the General Public

For other members of the public, without direct contact with COVID-19 cases, mental health may be affected by preventative measures too. For example, the initiation of social distancing, self-isolation and lockdowns limits face-to-face social contact with others; a model of interaction known to reduce the risk of Depressive disorder.^9^

Social connection is vital to well-being in humans^10^, and whilst internet-based media and applications such as Zoom, Skype, WhatsApp and FaceTime may allow for social interactions to continue, they do not replace the need for in-person human contact. It is possible that people may begin to experience transient mild to moderate depressive symptoms in the current circumstances. Additionally, there is likely to be increased vigilance towards cleanliness and hygiene, which when coupled with fears of infection and disease may lead to feeling more anxious than usual, and in persons with pre-existing psychiatric vulnerabilities it may manifest as the development of anxiety disorders.

In addition to heightened risk of Depression and Anxiety for the general public, it has been predicted that the COVID-19 pandemic is likely to increase rates of substance use, loneliness, domestic violence and child abuse^11^. Substance use may increase for those seeking recreation, thrill-seeking or as a way of self-medicating for stress and anxiety. Domestic violence and child abuse may increase due to isolation and lockdowns whereby abusers and victims will be in close proximity without much possibility of escape. These are all factors known to contribute to the development and worsening of psychiatric disorders.

### Impact on Patients of COVID-19

This is an area for further research. As yet there are no empirical data available relating to the mental health impact of contracting COVID-19. However, lessons from previous pandemic situations indicate that there is likely to be a risk of COVID-19 patients, who recover, developing symptoms of PTSD or Depression.^7^ This is likely to be moderated by personal traits such as resilience, availability and quality of social support and the patient’s own worries related to the illness and recovery.^8^

### Impact on Healthcare Workers

Whilst healthcare workers reported increased psychological distress during the SARS pandemic, perception of risk and perceptions of own health mediated the development of psychiatric morbidity.^12^ Those who perceived themselves to be at risk, were without protective gear and felt their own health to be sub-optimal, were more likely to develop psychiatric morbidity as compared to those who volunteered to work on SARS wards.^13^ This indicates the importance of organisational and governmental support for healthcare workers in terms of provision of suitable equipment, protective measures and working conditions to ensure minimal mental health difficulties. It also indicates that pre-existing factors such as psychological resilience, emotional resources such as social/familial support and preparedness may improve mental health outcomes for healthcare workers.

### Impact on Persons with Pre-Existing Mental Health Difficulties

Mental health conditions are chronic health co-morbidities that are often overlooked and considered comparatively unimportant as compared to medical co-morbidities like diabetes, hypertension etc. However, people suffering from mental health conditions already experience poverty, chronic medical problems and social disparity. Needless to say, COVID-19 is likely to adversely affect this population more acutely than the rest.^14^

For persons with pre-existing psychiatric disorders, the main concern is the worsening of symptoms; for those with pre-existing vulnerabilities, it is the onset of psychiatric symptoms that is the primary concern. Stress is a well-established contributor to development, onset and severity of mental health disorders.^15^ Anxiety and Depression are on the rise in the general population, with one in three people experiencing anxiety and one in five sleep disturbance and depressive symptoms.^16^ Sufferers of generalised anxiety and health anxiety may find increase in their symptoms during pandemic. Depressive symptoms may worsen with increased low mood, decreased energy, and limited interest in day-to-day activities. In crises, fear intensifies symptoms in people with pre-existing mental health disorders.^17^ For instance, stress is a well-documented factor in the worsening of symptoms of people with Schizophrenia and Bipolar Affective Disorder. In the aftermath of a natural disaster, patients suffering from Schizophrenia show the highest avoidance and have low approach coping, followed by patients suffering from Bipolar Disorder.^18^ It is possible that those with Obsessive-Compulsive Disorder (OCD) with pre-occupations of contamination and cleanliness may experience increased frequency and intensity of obsessive thoughts. The emphasis on frequent hand washing and the risk of infection after touching objects and meeting people as a part of COVID-19 preventions, as propagated by the government, media and social media, enhances the risk of relapse in persons with OCD.^19^

### Possible General Interventions

In the current era of international travel, any outbreak of infection has the potential to become a pandemic, leading to consequences for world health, economics and politics. Pandemics create a burden on healthcare professionals because of increased workload, high absenteeism, high mortality rates, lack of personal protective equipment, risk of infection and transmitting infection to loved ones, and ethical dilemmas of providing care to people.^20^

### Resilience Training

As seen in previous such crises (SARS 2003, Influenza 2009), healthcare workers who were adequately trained to deal with disaster situations reported better coping and no long term psychiatric problems as compared to people with no prior training.^21^ Therefore, the introduction of resilience training for healthcare professionals may be beneficial. Resilience training leads to professionals being better able to cope with highly stressful situations.^22^

Resilience training has 10 steps: 1-Family-work balance, 2. Antiviral prophylaxis, 3. Need for reliable, consistent, and timely information, 4. Education and preparation of employees’ families and the community, 5. Ethical concerns and fairness, 6. Visibility and presence of leadership, 7. Valuing the contributions of frontline staff, 8. Addressing mistrust or fear of health care workers, 9. Need for more information about how staff may be redeployed to unusual duties/work areas, 10. Need for ongoing resilience training.^22^

### Social Media Use

As fear is usually higher than risk of infection^23^, it is important for people to maintain a sense of normalcy in times of social distancing and quarantine. Any news, verified or not, via social media and television adds to the panic and fears of the public. In order to mitigate this, the public should be encouraged to decrease or minimise excess exposure to social media. Beneficial use of social media such as to communicate with loved ones, provide support and reassurance, provide practical advice and to seek help in case of a mental health difficulty can be used to support the public and pre-empt panic.^23^

### Social Connection & Individual Actions

Due to mass quarantine and social distancing, to prevent a sense of disconnection, people are advised to communicate with loved ones, reduce exposure to the news, maintain a positive attitude, take good care of their health, be aware of their own emotions and talk to someone if feeling sad or anxious. It is also advisable to rely on official news outlets and government guidelines regarding the pandemic.^24^

### Tele-Health

It has been suggested that psychiatric morbidities in developing countries may be mitigated by the provision of tele-health services, task-sharing amongst professionals and guidance and advice from professionals in more development nations.^25^ Whilst these measures would offer some respite, there are certain caveats such as limited internet access for many, the limited number of mental health professionals available in non-urban areas, and privacy, confidentiality and risk management issues with tele-health.

### Possible Mental Health Specific Interventions

In order to address mental health distress, steps may be taken to ensure psychological and emotional wellbeing. A comprehensive, multidisciplinary plan involving psychiatrists, nursing staff, and medical and emergency teams who work at the frontline may be established at organisational level. This will allow the incorporation of psychiatric interventions in the management of patients with coronavirus and their families.^20^,^26^ Clear and consistent information regarding the disease and management plan should be provided to everyone so as to avoid panic and confusion.^27^ As we try to limit exposure for the public and healthcare workers, the already strained frontline workers are left to deal with the psychological stress of patients. It is essential to offer tele-psychiatry in all medical settings to deal with the psychological burden of the disease.^25^ Furthermore, institutional guidelines need to be created to address how to deal with mental health difficulties in patients, healthcare workers and the general population, and to ensure the availability of specialist care.^26^

Telemedicine services are readily available to anyone owning a telephone or a computer^28^,^25^ and can be provided through video calls, phone call, text messaging and online forums. In China, the government has set up telemedicine units to give appropriate guidance, spread awareness and provide psychological counselling to those in need of it in order to prevent the long term effects of mental health distress.^27^,^25^

### Psychological First Aid

Psychological First Aid is a psychological intervention offered to survivors of disasters or disease outbreaks such as that of COVID-19. It emphasises compassion for survivors, the provision of informational care and creating space for listening to their stories, in order to ease their early rehabilitation.^29^ In crises, it is important to provide safety, adequate information, basic needs and comfort.^29^ In the current circumstances, where fear, anxiety or ‘pandemic anxiety’ may make one feel out of control, psychological first aid can be a helpful way to aid people in managing their experiences and emotions.

## CONCLUSIONS

The coronavirus outbreak in Wuhan, China, in December 2019 and the resulting global pandemic has caught the world unprepared to deal with an infection of this magnitude. In addition to the relatively high mortality rates, COVID-19 has caused widespread psychiatric distress that may lead to long term concerns such as PTSD, Depression, Anxiety and worsening of pre-existing psychiatric disorders. A comprehensive psychiatric response is needed. With the drastic increases in stress, workload and uncertainty for healthcare workers, the general public and people with mental health conditions, a focus on mental health management is essential. Early and timely interventions may stave off a mental health crisis.

Whilst interventions are possible and are likely to be helpful, this situation also highlights areas that need development. For instance, training for pandemic crises should be standard procedure in health settings. Institutes should have plans of action for various crisis situations. Staff should be aware of necessary procedures and should maintain written records of such necessary procedures. Resilience training and training focused on the health and psychological well-being of healthcare staff should be routine practice. These steps are likely to help in the management of future crises, should they arise. In summation, it is likely that the COVID-19 crisis will have both short term and long term mental health consequences for the general population, healthcare workers and patients alike.

### Authors’ Contribution

**IIH:** Conceived the review, initiated the literature search, contributed to its draft and coordination.

**FT:** Conducted literature review, contributed to writing & reviewing the manuscript.

**SMT:** Conducted literature review, and contributed to write-up & critical revision.

All authors read and approved the final manuscript.
